# Dehydroabietic Acid Isolated from *Commiphora opobalsamum* Causes Endothelium-Dependent Relaxation of Pulmonary Artery via PI3K/Akt-eNOS Signaling Pathway

**DOI:** 10.3390/molecules19068503

**Published:** 2014-06-23

**Authors:** Wenyan Gao, Xiaoyan Dong, Nan Xie, Chunlan Zhou, Yuhua Fan, Guoyou Chen, Yanming Wang, Taiming Wei, Daling Zhu

**Affiliations:** 1 College of Pharmacy, Harbin Medical University-Daqing, Daqing 163319, Heilongjiang, China; E-Mails: gaowenyan1987@126.com (W.G.); remembertry@163.com (X.D.); zhouchunlan490326@163.com (C.Z.); fyh198306@126.com (Y.F.); cgy20050101@126.com (G.C.); wymingming@163.com (Y.W.); dalingzhu2000@163.com (D.Z.); 2 Biopharmaceutical Institute of the Heilongjiang Academy of Medical Sciences, Harbin 150081, Heilongjiang, China; 3 College of Pharmacy, Harbin University of Commerce, Harbin 150028, Heilongjiang, China; E-Mail: xienan2002@126.com

**Keywords:** *Commiphora opobalsamum*, dehydroabietic acid, sandaracopimaric acid, nitric oxide, vasodilation, pulmonary artery

## Abstract

*Commiphora opobalsamum* is a Traditional Chinese Medicine used to treat traumatic injury, mainly by relaxing blood vessels. In this study, two diterpenes, dehydroabietic acid (**DA**) and sandaracopimaric acid (**SA**) were obtained from it by a bioassay-guided approach using isolated rat pulmonary artery rings. The structures of the two compounds were elucidated by spectroscopic methods (IR, ^1^H- and ^13^C-NMR, HR-ESI-MS). Both **DA** and **SA** reduced the contraction of phenylephrine-induced pulmonary arteries in a concentration-dependent manner, and endothelium contributed greatly to the vasodilatory effect of **DA**. This effect of **DA** was attenuated by N^G^-Nitro-L-arginine methyl ester (L-NAME, an eNOS inhibitor). Meanwhile, **DA** increased nitric oxide (NO) production, along with the increase of phosphorylation level of eNOS and Akt in endothelial cells. LY294002 (a PI3K inhibitor) could reverse this effect, which suggested the endothelial PI3K/Akt pathway involved in the mechanism underlying **DA**-induced relaxation of pulmonary artery. This work provided evidence of vasorelaxant substances in *Commiphora opobalsamum* and validated that PI3K/Akt-eNOS pathway was associated with **DA**-induced pulmonary artery vasodilation.

## 1. Introduction

Pulmonary arterial hypertension (PAH) is defined as a disease characterized by vasoconstricted and remodeled pulmonary arteries, which affects the pulmonary vasculature and increases pulmonary vascular resistance [1]. Although current therapies could improve the quality of life of PAH patients, PAH cannot be cured completely by the drugs used in current clinic therapy [2]. Recently, discovery for vasorelaxant compounds from natural products was of high interest regarding prevention and treatment of many vascular diseases. Phytochemical studies of *Commiphora opobalsamum* (*C. opobalsamum*) revealed the presence of flavonoids, sterols, triterpenes, saponins, volatilebases, and volatileoil [3]. It possesses extensive biological activities, such as antitumor, antiulcerogenic, hepatoprotective and hypotensive activities [4,5,6,7]. The aim of our research was to seek for potential vasorelaxant components on pulmonary artery in *C. opobalsamum*. The approach of bioassay-guided isolation was applied for this purpose, which is effective to search for vasorelaxant compounds from natural products as demonstrated by previous studies [8].

**Figure 1:**
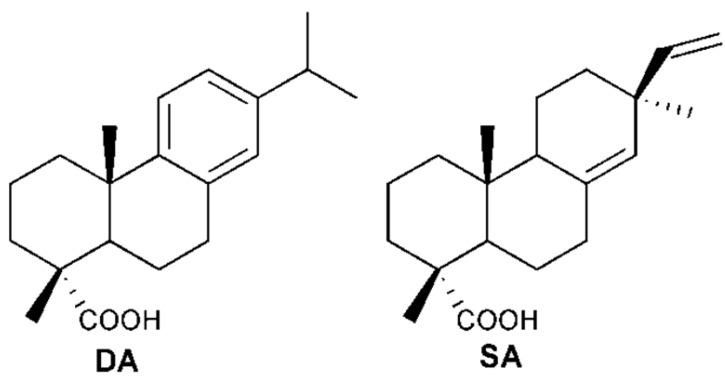
The chemical structures of **DA** and **SA**.

In the present study, dehydroabietic acid (**DA**) and sandaracopimaric acid (**SA**) were identified as relaxing pulmonary artery ingredients from *C. opobalsamum* (Figure 1). Previous reports have confirmed 15-pimaradien-3β-ol, a optical isomer of **SA**, could resist phenylephrine (PE)-induced contraction of the aorta, and its underlying mechanism involved extracellular Ca^2+^ influx blockade and activation of NO-cGMP pathway [9,10,11]. The vasorelaxant effect of **DA** was explored on pulmonary artery in this study. And the relaxation of **DA** on the pulmonary artery could be diminished by L-NAME, implying that **DA** relaxed pulmonary artery through endothelial NO pathway. Further experiments validated the **PI3K/Akt-eNOS** signaling pathway was involved in its vasorelaxant effect.

## 2. Results and Discussion

### 2.1. Bioassay-Guided Isolation

**Figure 2:**
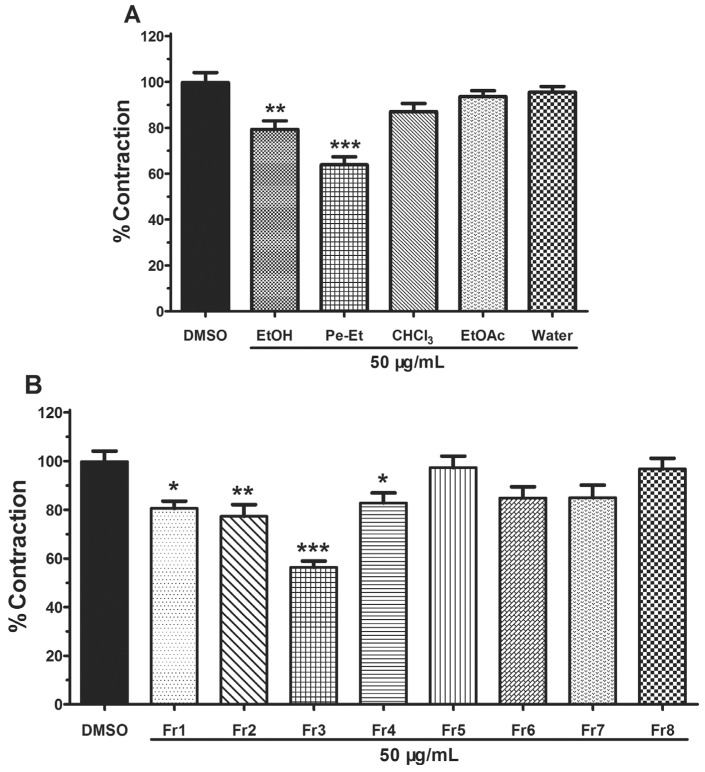
Vasorelaxant effect of all parts from *C. opobalsamum* on the PE-induced constriction of rat pulmonary artery. (**A**) Vascular effect of EtOH extract (EtOH) and four partitions [petroleum ether-soluble (Pe-Et), chloroform-soluble (CHCl_3_), EtOAc-soluble (EtOAc) and water-soluble (Water)] obtained from that extract. (**B**) Eight fractions ofpetroleum ether-soluble. Results are presented as means ± SEM, *n* = 5. * *p* < 0.05, ** *p* < 0.01 and *** *p* < 0.001 compared to vehicle (DMSO).

The bioassay-guided isolation results showed that the petroleum ether-soluble extracts could significantly relax PE-induced pulmonary artery rings at the concentration of 50 μg/mL, with 63.90% ± 7.78% contraction compared with vehicle control (DMSO) (Figure 2A). Each of the eight primary fractions (Fr1–Fr8) was also assessed using pulmonary artery rings (Figure 2B). At the same concentration (50 μg/mL), Fr1–4 could significantly inhibit the PE-induced contraction of pulmonary artery, especially Fr3, with the inhibition rate of 56.34% ± 5.82%. With further separation by a silica gel column, compounds **1** and **2** were obtained from Fr3.

### 2.2. Elucidation of Compounds’ Structures

Compound **1** was obtained as a white powder, while compound **2** was obtained in the form of white needle-like crystals when recrystallised from EtOAc. For both compounds, a total of 20 carbons, including a carbonyl group, were found in the ^13^C-NMR spectrum, and a strong characteristic C=O peak (1,693 cm^−1^) appeared in the IR spectrum. There were four carbon double bond signals in compound **1** in the ^13^C-NMR spectrum, with a molecular ion peak [M − H]^−^ at 301.6214 in the HR-ESI-MS, and six carbon double bond signals in compound **2**, with a molecular ion peak [M − H]^−^ at 299.6214. The optical rotations of compound **1** {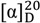: +2.0° (*c* 0.10 EtOH)} and **2**{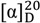: +63.0° (c 0.10 EtOH)} were in accordance with previous reported data [12]. By comparing their ^1^H- and ^13^C-NMR data with that of previous studies [13,14], compounds **1** and **2** were identified as **SA** and **DA**, respectively (Figure 1).

### 2.3. Effect of DA and SA on Pulmonary Arteries

**Figure 3:**
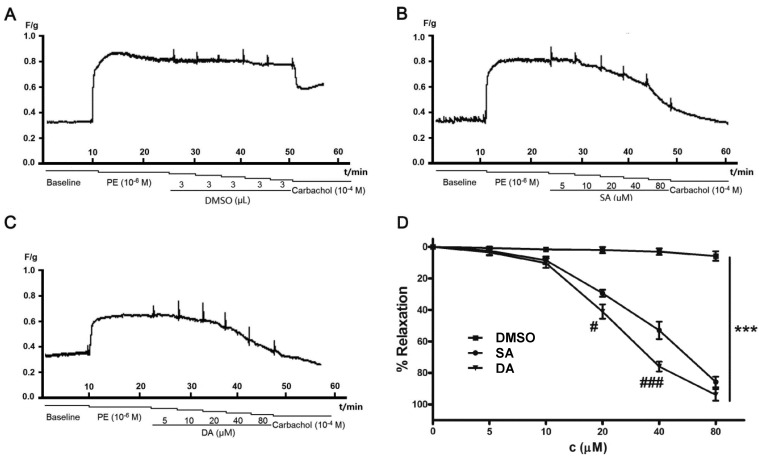
Effect of **DA** and **SA** on rats pulmonary artery rings precontracted with PE (10^−6^ M). The representative recorded curves of tension were presented as **A**–**C**, and **D** was the analysis results. Cumulative dosing of vehicle (DMSO) had no effect on rat pulmonary artery rings (**A**), however, dilatation effect of **SA** and **DA** on precontracted rat pulmonary artery rings were concentration-dependent (**B**,**C**). The results are represented as the means ± SEM (*n* = 5), *** *p* < 0.001 compared to corresponding control (DMSO), ^#^ *p* < 0.05 and ^###^ *p* < 0.001 showed the difference between **DA** and **SA** at the same concentration.

Pulmonary artery rings were pretreated with PE (10^−6^ M), and then **DA** or **SA** (5, 10, 20, 40, 80 μM) was accumulatively added to the bath. As shown in Figure 3, we found that both **SA** and **DA** were effective against PE-induced contraction of pulmonary artery rings in a concentration-dependent manner.

**SA** (5 μM) had a slight effect on the rest tone of the rings; the relaxation was only 2.46% ± 2.59%. However, when pulmonary artery rings were exposed to **SA** (20 μM), this effect was largely improved to 29.42% ± 2.25%. At a concentration of 80 μM, PE-induced contraction was suppressed by 85.75% ± 3.37%, which was close to the state of the pulmonary artery rings before being exposed to PE. The vasodilatory effect of **DA** was similar to that of **SA**, however, it was superior to that provided by **SA**, and EC_50_ value of **DA** and **SA** was 35.81 and 43.93 μM, respectively. **DA** and **SA** are both diterpenoids with a phenanthrene structure, but the vasodilatory effect of **DA** on the pulmonary artery was greater than that of **SA** at middle concentration (20–40 μM). The aromatic ring of **DA** may contribute to its effectiveness.

### 2.4. Effect of Endothelium and L-NAME on DA-Induced Vasorelaxation

**Figure 4:**
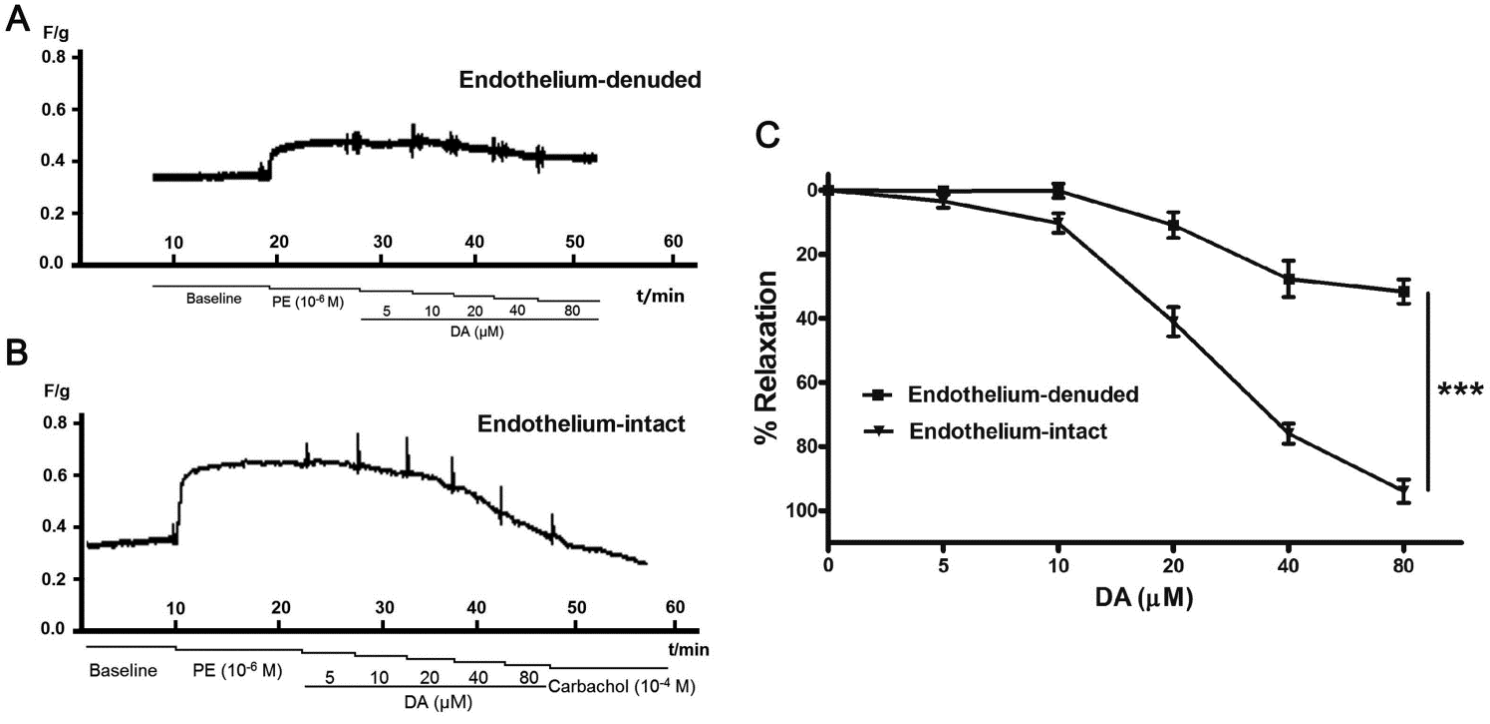
Effect of **DA** on endothelium-intact and endothelium-denuded rat pulmonary artery. Cumulative dosing of **DA** vasorelaxant effect on both endothelium-intact and endothelium-denuded rats pulmonary artery rings were detected (**A**,**B**). The results are represented as the means ± SEM (*n* = 5) (**C**), *** *p* < 0.001 for subdued effect of endothelium-denuded *versus* endothelium-intact group.

To evaluate the involvement of endothelium in **DA**-induced vasorelaxation, we examined the vasodilatory effect of **DA** on endothelium-denuded pulmonary artery rings. As shown in Figure 4, when the endothelium of pulmonary artery rings was removed, the vasodilatory effect of **DA** on the pulmonary artery decreased distinctly compared with that of the endothelium-intact rings. The relaxation ratios were only 31.62% ± 3.75% at the concentration of 80 μM, less than that of endothelium-inract (relaxation ratios of 93.94% ± 3.69%). This finding suggested that the vasodilatory effect of **DA** was closely related to the endothelium. And additional vasodilatory effects may be the result of the open calcium-sensitive potassium ion channels induced by DA [15].

**Figure 5:**
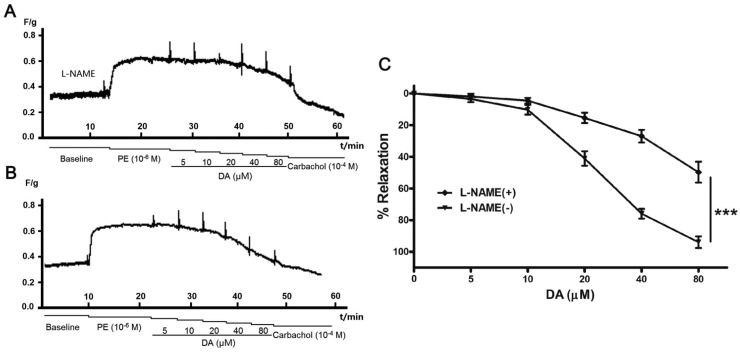
Effect of NO in DA-induced pulmonary artery rings relaxation. L-NAME (10^−4^ M) significantly reduced DA-induced dilation effect on endothelium-intact artery rings (**A**,**B**). Results were expressed as mean ± SEM (*n* = 5) (**C**), *** *p* < 0.01 for inhibitory effect of L-NAME *versus* DA group.

The fact that **DA** showed relaxant effect on endothelium-dependent manner suggests that NO pathways maybe involved in the response [16]. NO is a crucial mediator in endothelial vasodilator function, which is synthesized from the terminal guanidino nitrogen of L-arginine by NOS enzymes [17]. Substantial evidence has shown that NO-mediated relaxation of several substances or plant extracts in rat aortic rings [18,19,20], so we decided to investigate the involvement of endothelial NOS (eNOS) in **DA**-induced pulmonary artery rings relaxation. Pulmonary artery rings were pretreated with L-NAME (10^−4^ M) for 30 min before being pre-contracted with PE. As illustrated in Figure 5, as NO-induced vasorelaxation was blocked by L-NAME, DA-induced vasodilatation was diminished by about 50% at the concentration of 80 μM. This suggested that the mechanism underlying **DA**-induced relaxation depended on eNOS activity. Why carbachol caused a profound relaxation vasodilatory (Figure 5A) may be that the activity of carbachol was so strong that its effect couldn’t be completely blocked by L-NAME (100 μM) [21].

### 2.5. DA Promotes NO Release of PAECs

**Figure 6:**
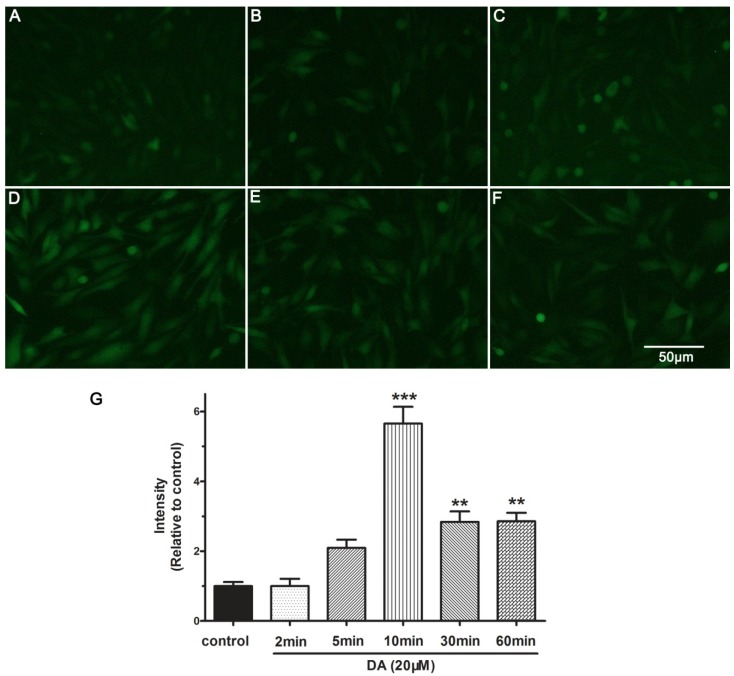
**DA**-induced NO production in PAECs within 60 min. NO production was visualized with fluorescence intensity. PAECs were treated with **DA** for different periods (**A**) 0 min (control); (**B**) 2 min; (**C**) 5 min; (**D**) 10 min; (**E**) 30 min; (**F**) 60 min. Results are presented as the mean ± SEM (*n* = 5) (**G**). ** *p* < 0.01 and *** *p* < 0.001 compared with the control group.

To determine whether NO release indeed is modulated by **DA** (20 μM), the intracellular NO production from 0 min to 60 min in PAECs was measured using DAF-FM DA fluorescence indicator. As shown in Figure 6A–F, DAF-FM DA fluorescence intensity was significantly increased from 2 min to 10 min, especially at 10 min, 5.65-fold compared with that at 0 min (control) (Figure 6D,G). However, the relative intensity of DAF-FM DA fluorescence rapidly decreased to 2.8-fold at 30 min post- exposure, and sustained to 60 min at this proximity level. The results indicated that **DA** stimulates NO release by achieving the maximum effect at 10 min. These demonstrated that **DA** might be a potent stimulus for eNOS activation in PAECs and took effect fast.

### 2.6. Role of the PI3K/Akt Pathway in DA-Induced Relaxation

**Figure 7:**
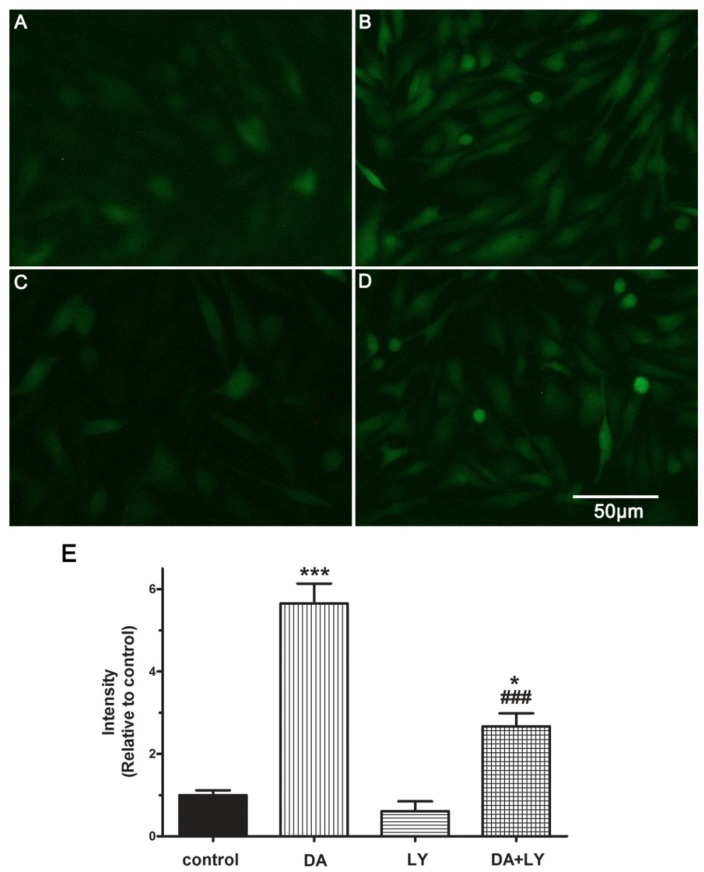
**DA** (20 μM) stimulated NO production in PAECs via PI3K/Akt pathway in 10 min. **DA** stimulated the release of NO product (**B**) and LY294002 (LY 10^−6^ M) reduced NO production in PAECs (**C**) compared with the control group (**A**). However, LY could also reduce the stimulation of **DA** on NO production significantly (**D**). Results are presented as the mean ± SEM (*n* = 5) (**E**). * *p* < 0.05 and *** *p* < 0.001 compared with the control group. ^###^ *p* < 0.001 *versus* **DA** group.

Previous studies indicated that the activation of the PI3K/Akt pathway could promote eNOS phosphorylation leading to NO-induced relaxations [22,23]. To explore whether **DA** affects the PI3K/Akt pathway, we blocked the PI3K/Akt signaling pathway with LY294002 (LY, 10^−6^ M, Figure 7). The fluorescence intensity was increased by 5.65-fold compared with the control group after treatment with **DA** (20 μM) in 10 min. After the cells were exposed to LY for 10 min, the fluorescence intensity was reduced by 0.9-fold compared with the control group. When the PAECs were exposed to LY plus **DA**, the fluorescence intensity was decreased to 2.67-fold of control, significantly reduced compared with the **DA** group (Figure 7B,D,F, *p* < 0.01). The experimental data thus showed that PI3K/Akt inhibitor LY could reduce NO production enhanced by DA, which suggested that the PI3K/Akt pathway plays an important role in the NO synthesis.

**Figure 8:**
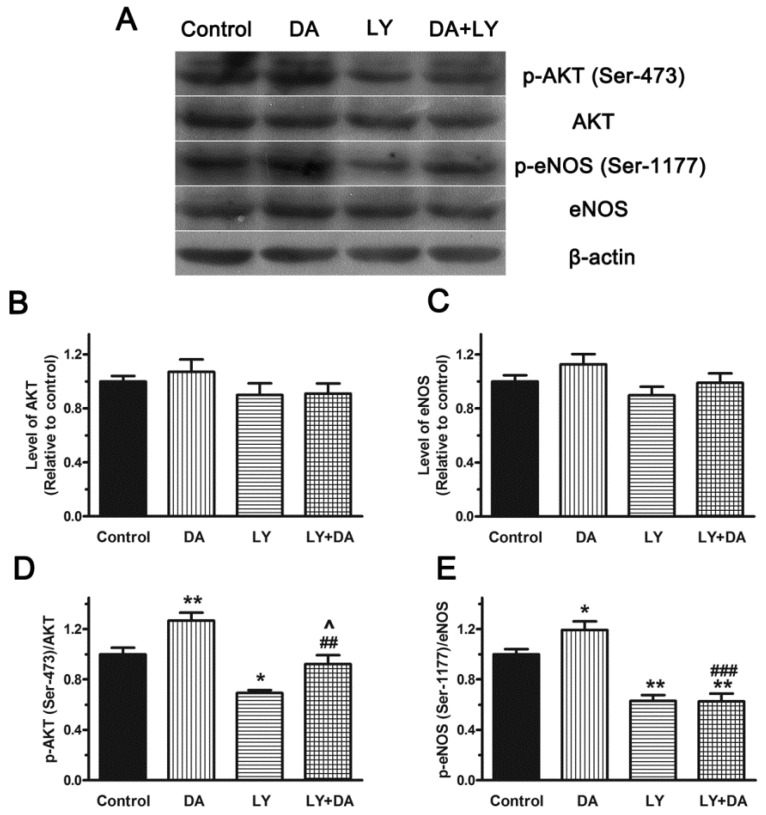
Role of the PI3K/Akt Pathway in **DA**-induced phosphorylation of AKT and eNOS in PAECs. PAECs were incubated with vehicle (DMSO), **DA** (20 μM), LY294002 (LY, 10^−6^ M) and **DA** (20 μM) plus LY (10^−6^ M) for 10 min. Then the levels of p-Akt (Ser-473), Akt, p-eNOS (Ser-1177) and eNOS (**A**) were determined by Western blot analysis. Results are presented as means ± SEM (*n* = 3) (**B**, **C**, **D**, **E**). * *p* < 0.05 and ** *p* < 0.01 *versus* control. **^##^** *p* < 0.01 and **^###^** *p* < 0.001 compared to corresponding **DA** group. **^** *p* < 0.05 compared to corresponding **LY** group.

In parallel, to better characterize the PI3K/AKT pathway involved in eNOS activation in response to **DA**, the expression of eNOS and Akt, as well as the phosphorylation level of eNOS (p-eNOS) and Akt (p-AKT) was assessed in PAECs using western blot analysis. Phosphorylation of Ser-473 on Akt is simultaneous with Akt activation and it was the most widely reported phosphorylation site according to previous reports [24,25]. Akt is an important determinant of eNOS phosphorylation at Ser-1177, leading to vascular smooth muscle relaxation [26]. So we firstly chose these phosphorylation sites for our study. Resullts showed that exposure of PAECs to **DA** (20 μM) in 10 min didn’t change the expression of Akt and eNOS (Figure 8B,C), but it caused the appearance of a strong phosphorylation signal of Akt (Ser-473) and eNOS (Ser-1177), and the values of Ser-473/AKT and Ser-1177/eNOS were 1.27 ± 0.11 and 1.19 ± 0.12 fold, respectively, compared with the corresponding control. When treated with LY (10^−6^ M) plus **DA** (20 μM), the value of Ser-473/AKT and Ser-1177/eNOS was reduced to 72.7% (*p* < 0.01) and 52.5% (*p* < 0.001) of control, respectively. It suggested that **DA** could activate eNOS through activation of the upstream PI3K/AKT signal pathway. However, the inhibitory effect of LY on the production of Ser-1177 was stronger than that of Ser-473, which implyed that other phosphorylation sites of AKT might also be activated by **DA**. All of these results indicated that phosphorylation activation of PI3K/Akt pathway is necessary for NO production induced by **DA**.

## 3. Experimental Section

### 3.1. General Information

The ^1^H-NMR and ^13^C-NMR spectra were recorded on a Bruker Avance 400 spectrometer (Bruker Company, Karlsruhe, Germany) in CDCl_3_ using tetramethylsilane (TMS) as an internal standard. The HR-ESI-MS data were obtained from a Waters LCT-Premier spectrometer (Waters Corporation, Milford, PA, USA) in ESI ionisation mode. The IR spectra were recorded on FTIR-8400S (Shimadzu Corporation, Kyoto, Japan). Optical rotations were determined with a WZZ-2B auto polarimeter (Shanghai Shenguang instrument Co. Ltd., Shanghai, China). Column chromatography was carried out on silica gel (200–400 mesh; Qingdao Haiyang Chemical Co. Ltd., Qingdao, China). Separations were monitored by TLC on GF_254_ plates (0.25 mm, Qingdao Haiyang Chemical Co. Ltd., Qingdao, China) and were visualized by UV inspection and or staining with 1% vanillin in concentrated sulphuric acid and heating. The tension data were recorded using the ALC-MPA force transducer (Shanghai Alcott Biotech Co, Ltd., Shanghai, China), which allowed eight rings to be recorded and analysed at the same time. The microscopic fluorescent images were taken by Nikon TE2000 Olympus fluorescent microscope (Nikon Corporation, Kyoto, Japan). Protein concentrations were measured with Bio-Rad protein assay kit (Bio-Rad Laboratories, Inc., Berkeley, CA, USA).

### 3.2. Materials and Reagents

The resin of *C. opobalsamum* was purchased in December 2011 from the AnGuo medicinal material market (Daqing, China). It was identified by Vice Professor Taiming Wei (Institute of Biological Pharmacy, Harbin Medical University, China). A voucher specimen (voucher number: ZY089) was deposited in the Institute of Biological Pharmacy, Harbin Medical University, China Herbarium. N^G^-Nitro-l-arginine Methyl Ester (l-NAME), fluorescent indicator 3-amino-4-aminomethyl-2',7'-difluorescein diacetate and LY294002 were purchased from Beyotime Institute of Biotechnology Co. Ltd. (Shanghai, China). Protein kinase B (Akt) antibody and phosphorylated Akt at Ser-473 (p-Akt) antibody, eNOS antibody and phosphorylated eNOS at Ser-1177 (p-eNOS) antibody were obtained from Cell Signaling Technology, Inc. (Danvers, MA, USA). All other reagents were from common commercial sources.

### 3.3. Extraction and Isolation

A bioassay-guided isolation approach was carried out following the earlier reported method [8]. The dried resin of *C opobalsamum* (4.0 kg) was exhaustively extracted with one volume of 95% EtOH under reflux (3 × 2 h). After removing the solvent under vacuum, the residue (722.0 g) was suspended in H_2_O and extracted successively with petroleum ether, CHCl_3_ and EtOAc to yield petroleum ether-soluble, CHCl_3_-soluble and EtOAc-soluble fractions. As the petroleum ether-soluble (112.3 g) fraction presented obvious PE-induced contraction activity on the pulmonary artery, this fraction was subjected to column chromatography on silica gel (4.0 kg) with a petroleum ether/ethyl acetate gradient (1:0 to 0:1, v/v). This generated eight fractions (Fr1–8) on the basis of the TLC profiles. The effects against PE-induced contraction of the pulmonary artery of all of the fractions were determined, and Fr3 showed a significant effect. Fr3 (16.9 g) was separated by silica gel column chromatography [petroleum ether–EtOAc (20:1, 10:1,4:1, 2:1, v/v)] to isolate compounds **1** (342 mg) and **2** (215 mg). Their structures were elucidated on the basis of spectroscopic methods (IR, ^1^H- and ^13^C-NMR, HR-ESI-MS).

### 3.4. Vessel Rings Preparation

The male Wistar rats (180–220 g) used in this study were treated as verified by the Institutional Animal Care and Use Committee (IACUC) of Harbin Medical University. According to previous literature [27], the rats were killed rapidly, and their mesentery and pulmonary arteries were quickly removed, cleaned of adherent connective tissues and cut into 3-mm-length rings under a dissecting microscope. The artery rings were maintained in ice-bathed Krebs solution containing, in mM, NaCl, 118.0; KCl, 4.7; KH_2_PO_4_, 1.2; MgSO_4_, 1.2; NaHCO_3_, 15.0; glucose, 5.5; and CaCl_2_, 2.5 (pH 7.4) throughout the whole process. Endothelium was damaged mechanically by a thin wire when preparing the endothelium-denuded rings. The endothelial integrity was assessed qualitatively by the degree of relaxation caused by carbachol (10^−4^ M) followed by treatment with phenylephrine (PE, 10^−6^ M). The rings were regarded as endothelium-denuded if they displayed less than 10% relaxation.

### 3.5. Effect of All the Parts on Pulmonary Artery Rings

All the fractions and compounds were dissolved in dimethyl sulfoxide (DMSO). PE and carbachol were dissolved in ultrapure water. Each artery ring was fixed on a force transducer and placed in a water-jacketed organ bath filled with 3 mL of Krebs solution and exposed to O_2_. The rings were initially stretched with a basal tension of 0.30 g for 30 min to achieve a state of equilibrium. After the vessel rings were contracted with 3 μL of PE for 15 min, the drugs (3 μL every time) were added to water-jacketed organ baths, and the tensions of each force transducer were recorded. The concentrations of the drugs were increased at 5 min increments. Carbachol (10^−4^ M) was used to detect the vasodilatory activity of the pulmonary artery after treated with drugs. Following are the formulas for the calculations of %contraction and %relaxation:

% Contraction = (F − F_0_)/(F_PE_ − F_0_) (1)

% Relaxation = 1 − % Contraction
(2)

F: Tension of the rings treated with drugs. F_0_: Pre-load. F_PE_: Tension of the rings treated with PE. (1): %Contraction (PE 10^−6^ M) = 100%; (2): %Relaxation (PE 10^−6^ M) = 0%.

### 3.6. Culture of Pulmonary Artery Endothelial Cells (PAECs)

PAECs were isolated from neonatal bovine obtained from a local abattoir. The calves’ use was in full compliance with the rules of the Ethics Committee of Laboratory Animals. PAECs were cultured in culture medium containing 20% fetal calf serum, penicillin (100 U/mL) and streptomycin (100 U/mL), supplemented with L-glutamine in 5% CO_2_ at 37 °C on culture flasks as previously described [28]. When the cells reached sub-confluence, they were pretreated with culture medium containing different concentrations of drugs that were tested in the experiments.

### 3.7. Measurement of NO Production in PAECs

DAF-FM DA as a fluorescence indicator was used to detect intracellular NO [29]. When PAECs grown on a microcoverglass of the 3 cm culture dish reached 80% confluence, cells were exposed to **DA** (20 μM) for 0 min, 2 min, 5 min, 10 min, 30 min and 60 min, respectively, then washed three times with phosphate-buffered (PBS, pH 7.4). After loading with 5 mM DAF-FM DA at 37 °C for 20 min in dark, the PAECs were rinsed three times with PBS and maintained in PBS throughout the experiments. The microscopic fluorescent images were taken by an Olympus fluorescent microscope at 495 nm (excitation) and 515 nm (emission) and analyzed with Image-Pro Plus software (Version 5.0). The average fluorescent density was measured to index the NO level [30].

### 3.8. Western Blotting

The expressions of nitric oxide synthase (eNOS) and protein kinase B (Akt), as well as the phosphorylation level of eNOS (Ser-1177) and Akt (Ser-473) in PAECs were assessed using western blotting as previously reported [25,31]. Briefly, after treatment with drugs, cells were washed three times with cold PBS and then lysed with alysis buffer (Tris 50 mM, pH 7.4, NaCl 150 mM, Triton X-100 1%, EDTA 1 mM and PMSF 2 mM) on ice. Lysates were sonicated for 1 min and then supernatant was collected by centrifugation at 13,500 rpm for 15 min at 4 °C. Total proteins (20 mg) were separated by SDS-PAGE and transferred to nitrocellulose membranes. After blocked with a blocking buffer containing 5% skimmed milk, the membranes were then incubated with the appropriate antibodies as follows: eNOS (1:1000), Akt (1:500), anti-phospho-Akt (Ser-473, 1:500), and anti-phospho-eNOS (Ser-1177, 1:1000). After maintained at 4 °C overnight, membranes were incubated with the secondary antibody (goat anti-rat IgG or goat anti-rabbit IgG) for 1 h at room temperature. Then the membranes were incubated with ECL and exposed to X-ray film in dark. Densitometric analyses were performed with the Bio-Rad Quality-One software.

### 3.9. Statistical Analysis

The data were expressed as the means ± SEM. Two-way ANOVA followed by Dunnett’s test was performed in the statistical analysis to study the effect of endothelium and L-NAME on DA-induced vasorelaxation and one-way ANOVA was used in other sections. A probability level of *p* < 0.05 was regarded as significantly different.

## 4. Conclusions

In conclusion, our study first found that **DA** and **SA** are the main constituents of *C. opobalsamum* that exhibits vascular effects, which may be regarded as an index for evaluating the quality of *C. opobalsamum* for blood stagnation. Both **DA** and **SA** could relax PE contracted pulmonary artery in a concentration-dependent manner. **DA**-induced vasodilation of pulmonary artery was endothelium-dependent and in accordance with the release of NO. It might be mediated by the activation of PI3K/AKT signaling pathway in endothelium.
